# Sensitization and translocation of TRPV1 by insulin and IGF-I

**DOI:** 10.1186/1744-8069-1-17

**Published:** 2005-04-27

**Authors:** Jeremy J Van Buren, Satyanarayan Bhat, Rebecca Rotello, Mary E Pauza, Louis S Premkumar

**Affiliations:** 1 Department of Pharmacology, Southern Illinois University School of Medicine Springfield, IL 62702, USA; 2 Department of Medical Microbiology and Immunology, Southern Illinois University School of Medicine Springfield, IL 62702, USA; 3 Department of Internal Medicine, Southern Illinois University School of Medicine Springfield, IL 62702, USA

**Keywords:** insulin, IGF-I, diabetes, TRPV1, capsaicin, vanilloid

## Abstract

Insulin and insulin-like growth factors (IGFs) maintain vital neuronal functions. Absolute or functional deficiencies of insulin or IGF-I may contribute to neuronal and vascular complications associated with diabetes. Vanilloid receptor 1 (also called TRPV1) is an ion channel that mediates inflammatory thermal nociception and is present on sensory neurons. Here we demonstrate that both insulin and IGF-I enhance TRPV1-mediated membrane currents in heterologous expression systems and cultured dorsal root ganglion neurons. Enhancement of membrane current results from both increased sensitivity of the receptor and translocation of TRPV1 from cytosol to plasma membrane. Receptor tyrosine kinases trigger a signaling cascade leading to activation of phosphatidylinositol 3-kinase (PI(3)K) and protein kinase C (PKC)-mediated phosphorylation of TRPV1, which is found to be essential for the potentiation. These findings establish a link between the insulin family of trophic factors and vanilloid receptors.

## Introduction

TRPV1 is a Ca^2+ ^permeable nonspecific cation channel, located on peripheral sensory neurons, that serves as a molecular detector for heat, capsaicin, protons, and endovanilloids [1-4]. Moreover, its role as a heat sensor (activation threshold of ~43°C), its influence from trophic/inflammatory agents (i.e. nerve growth factor, bradykinin, prostaglandins, etc.) and its vasodilatory effect on small vessels (by releasing CGRP) make TRPV1 an essential component of the pain pathway [3,5-11].

In peripheral nerves, insulin does not promote its typical metabolic effects of glucose and amino acid uptake [12]. However, physiologic concentrations can act as neurotrophic factors, in combination with nerve growth factor (NGF), to stimulate neurite outgrowth and survival of both sensory and sympathetic neurons [13-15]. Furthermore, it has been proposed that insulin and IGFs exert trophic influences on the same neurons that are responsive to NGF by sharing common signaling pathways [13].

Though it is widely believed these neural disturbances are secondary to hyperglycemia [16], this remains controversial. Results from the Diabetes Control and Complications Trial show that intensive glycemic control for 5 years reduced the incidence of neuropathy by 60% in Type I patients [17]. However, the fact that strict glucose regulation does not completely prevent diabetic peripheral neuropathy suggests additional mechanisms as a result of insulin deficiency may be involved.

Experiments in humans show that non-diabetic, normoglycemic subjects have heat thresholds that correlate positively with insulin sensitivity [18]. Furthermore, circulating levels of IGFs and NGF are reduced in diabetes and to a greater degree in patients with thermal sensory deficits characteristic of neuropathy [19-21]. Growth factor replacement therapy improves neuronal regeneration [22,23] and insulin, under conditions of rapid infusion, can restore decreased neurogenic vasodilation [24,25]. Taken together, these studies suggest that insulin and trophic factor deficiencies may alter sensory nerve function [26].

In this study we demonstrate that insulin and IGF-I enhance TRPV1-mediated membrane currents by enhancing receptor sensitivity and translocation from cytosol to plasma membrane in heterologous expression systems and cultured dorsal root ganglion (DRG) neurons in a PKC-dependent manner. Preliminary results of this study have been published elsewhere in an abstract form [27].

## Materials and Methods

### Oocyte Electrophysiology

Oocytes were isolated from tricaine-anesthetized *Xenopus laevis *and separated from the follicular layer after incubation with collagenase (1–2 mg/ml). 3–7 days following cRNA injections (50–70 nl, 1 μg/μl), oocytes were stored at 18°C and used for experiments. Double electrode voltage clamp (-60 mV) was performed using a Warner amplifier (Warner Instruments, OC725C, Hamden, CT, USA) with 100% d.c. gain, digitized and stored on video tape. Experiments were performed at 21–23°C. Oocytes were placed in a Perspex chamber superfused (5–10 ml min^-1^) with Ca^2+^-free Ringer solution containing (in mM): 100 NaCl, 2.5 KCl, 5 HEPES, pH 7.35. Within figures, current traces were shown as initial response to agonist juxtaposed on currents recorded following incubation with insulin, IGF-I and various inhibitors. Time course experiments consisted of current recording during capsaicin application in the presence of either control (first 20 min) or insulin (last 15 min) containing solutions. In all experiments, fold increase was calculated as agonist induced current amplitude in the presence of trophic factors and inhibitors divided by the agonist induced initial current amplitude under control conditions before their bath application. I-V relationships were measured using 500-ms voltage ramps from -80 mV to +80 mV.

### DRG Culture and Electrophysiology

Dorsal root ganglia were isolated from embryonic day 18 (E18) rats, triturated and cultured for 5–7 d in Neurobasal/B-27 (Life Technologies; Grand Island, NY) + 10% fetal bovine serum (FBS) on poly-D-lysine-coated glass coverslips. For perforated patch recording, the bath solution contained (in mM): 140 NaGlu, 2.5 KCl, 10 HEPES, 2 MgCl_2_, 1 EGTA, pH 7.35 and the pipette solution contained (in mM): 130 NaGlu, 10 NaCl, 2.5 KCl, 10 HEPES, 1 MgCl_2_, 0.2 EGTA, and amphotericin B (240 μg/ml). For cell-attached recording, the bath solution contained (in mM): 140 Kglu, 10 NaCl, 10 HEPES, 1 EGTA, 2 MgCl_2_, pH 7.35 and the pipette contained (in mM): 140 NaGlu, 2.5 KCl, 10 HEPES, 2 MgCl_2_, pH 7.35. Currents were recorded using a WPC 100 patch clamp amplifier (E. S. F. Electronic, Goettingan, Germany) or Axopatch 200B (Axon Instruments, Union City, CA). Data were digitized (VR-10B, Instrutech Corp.; Great Neck, NY) and stored on video tape.

For analysis, data were filtered at 2.5 kHz (-3db frequency with an 8-pole low-pass Bessel filter, Warner Instruments, LPF-8) and digitized at 5 kHz. Data analyses were performed on continuous stretches greater than 20 s from patches that contained one or two channels. Single channel current amplitude and P_o _were calculated from all point amplitude histograms fitted with Gaussian functions (Microcal Origin; Northampton, MA).

### Ca^2+ ^Imaging

Cells were grown on glass coverslips, then incubated with 5 μM Fluo-4AM (Molecular Probes; Eugene, OR) for 20 min at 37°C, washed with physiological buffer [(in mM): 140 NaCl, 5 HEPES, 2 CaCl_2_, 1 MgCl_2_, 2.5 KCl, pH 7.35], and treated with 500 nM capsaicin +/- 1 μM insulin immediately prior to analysis by confocal microscopy (Fluoview, Olympus; Mellville, NY). Data for each cell was quantified as the fluorescence after treatment (F) divided by initial fluorescence (F_o_) at t = 0 (Fluoview software).

### Transfection of Cells and Cell Culture

HEK 293 cells, which endogenously express IGF-I receptors [29] were cultured in Dulbecco's modified Eagle's medium (high glucose) supplemented with 10% fetal bovine serum, 50 units/ml penicillin, and 50 μg/ml streptomycin (Invitrogen; Carlsbad, CA) and maintained under 95% air/ 5% CO_2 _at 37°C. Cells were transiently transfected with wild type TRPV1, TRPV1eGFP, TRPV1 S502/S800A mutant (gifted by D. Julius, N. Kadei, M. Tominaga, respectively), or TRPV1-V5-His tagged plasmid using Lipofectamine2000 reagent (Invitrogen) according to manufacturer's protocol. TRPV1-V5-His tagged plasmid was constructed in pcDNA3.1 vector using Topo Cloning kit (Invitrogen). To determine the membrane translocation of TRPV1, confocal images (1 μm sections) of GFP-tagged TRPV1 fluorescence was obtained. The intensity of the brightest membrane fluorescence was selected and quantified using MCID imaging software (Imaging Research Inc; St. Catherines, ONT)

### Determination of Surface TRPV1

After 36 h of TRPV1 transfection, the cells (grown on 100 mm tissue culture plate) were insulin-treated and biotinylated with membrane impermeable NHS-LC-biotin (1.5 mg/ml in PBS; Pierce, Rockford, IL) as per manufacturer's protocol. Labeled cells were lysed in RIPA buffer and immunoprecipitated overnight with 10 μl of goat anti-VR1 polyclonal antibody/ Protein A/G Plus-agarose (Santa Cruz Biotechnology, Santa Cruz, CA) following manufacturer's protocol. Immunoprecipitates were eluted with 2X SDS sample buffer, separated on 7.5% SDS-PAGE, and transferred to PVDF membrane. To detect total TRPV1, blots were probed with rabbit anti-VR1 polyclonal antibody (1:1000; Affinity Bioreagents, Golden, CO). Specific antibody binding was detected using HRP-conjugated anti-rabbit secondary antibody (1:20,000; Jackson Immunoresearch, West Grove, PA) and Super Signal reagent (Pierce). To detect surface TRPV1, blots were stripped and probed with neutravidin-HRP (1:20,000; Pierce). Chemiluminescence was captured in Hitachi CCD Bio Genetic Systems after exposing the blot to Super Signal reagent (Pierce). Data were quantitated using LabWorks analysis software (UVP Inc., Upland, CA) and surface TRPV1 was normalized using total TRPV1. TRPV1-V5-His transfected cells were biotinylated as above and purified using Ni-NTA agarose (Qiagen, Valencia, CA) as per manufacturer's protocol.

### Immunohistochemistry

Cells were plated on glass coverslips coated with 40 μg ml^-1 ^poly-L-lysine, treated with insulin/IGF/PDBu after 36 h of transfection, and fixed with 4% paraformaldehyde for 1 h. The cells were probed with rabbit anti-VR1 antibody (1:10,000; Affinity Bioreagents) followed by rhodamine red-X conjugated anti-rabbit secondary antibody (1:50; Jackson Immuno Research) following method described by Santa Cruz Biotechnology. Confocal images (1 μm sections) were captured using 570 nm laser. The brightest membrane fluorescence was selected from control and treated cells and quantified using MCID imaging software (Imaging Research Inc.).

Unless otherwise stated, all chemicals were obtained by Sigma (St. Louis, MO). Data are given as mean ± s.e.m. and statistical significance (set at P < .05) was evaluated using Student's *t*-test or one-way ANOVA.

## Results

### Insulin and IGF-I potentiate TRPV1 current in oocytes

**Figure 1:**
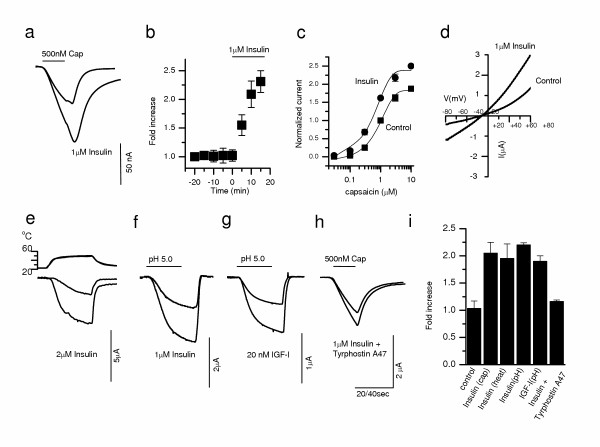
Insulin and IGF-I potentiate TRPV1 currents in *Xenopus *Oocytes. **a**, A representative dual-electrode whole-oocyte experiment showing potentiation of capsaicin induced TRPV1 currents following 15 min incubation of insulin (see Methods). **b**, Time course for capsaicin induced current (■: 500 nM capsaicin, 30 sec application) before and after insulin exposure at 5 (*P *< .05), 10 (*P *< .01), and 15 min (*P *< .01) incubation times (*n *= 7). **c**, Dose response curve of capsaicin before (squares) and after 5 μM insulin (circles) application, the currents were normalized to 1 μM capsaicin before insulin application (n = 2 to 4, before and after insulin). Both the sensitivity (EC_50 _shifted from 0.9 to 0.6 μM) and maximal response increased (1.8 to 2.4). **d**, Representative I-V relationships (1 s, 1 mV step ramp from -80 to +80 mV) before and after insulin treatment that demonstrates the outward rectification typical of TRPV1 channels. **e**, Potentiation of heat-induced currents by insulin. **f**, Potentiation of pH induced currents by insulin. **g**, Potentiation of pH induced currents by IGF-I. **h**, Tyrphostin A47, an IR / IGFR antagonist, blocked insulin potentiation. **i**, Summary graph showing fold increase in TRPV1 currents following 10 min incubation with control, insulin (cap: *n *= 5, *P *< .01; pH: *n *= 4, *P *< .01), IGF-I (*n *= 3, *P *< .01), and insulin + tyrophostin A47 (*n *= 3, *P *< .01). Results are expressed as increase in current amplitude relative to initial capsaicin or pH response.

First, we tested whether insulin and/or IGFs influence TRPV1-mediated membrane currents. The effect of insulin and IGF-I on cloned TRPV1 was characterized through dual-electrode voltage clamp (V_m _= -60 mV) of *Xenopus *oocytes injected with TRPV1 cRNA. Insulin (1 μM) significantly potentiated the response to capsaicin (500 nM) greater than 2 fold (Fig. 1a). The time course of insulin-induced potentiation of capsaicin response shows a significant increase in current amplitude for 5, 10 and 15 minute incubation periods compared to initial levels (Fig. 1b). Dose response curves of capsaicin show that both the sensitivity (EC_50 _shifted from 0.9 to 0.6 μM) and the maximal response (normalized to 1 μM capsaicin induced current before insulin application) increased (from 1.8 to 2.4) after incubation of oocytes with insulin (circles) for 5 min (Fig. 1c). A representative current-voltage relationship (I-V curve) confirms the outward rectification characteristic of TRPV1 current and shows that, in the presence of insulin, amplitude is increased at both positive and negative potentials (Fig. 1d). In addition, current potentiation was also seen with heat evoked responses (Fig. 1e) and protons (Fig. 1f), an endogenous TRPV1 agonist. These experiments show that insulin is increasing TRPV1-mediated currents and making the receptor more responsive to exogenous and endogenous activators.

Oocytes endogenously express insulin-like growth factor receptors (IGFR) rather than insulin receptors (IR) [30]. Insulin binds to IGFR with 100–1000 times lower affinity than IR. On the other hand, IGF-I binds to IGFR with much higher affinity, so we tested whether lower concentrations of IGF-I could potentiate TRPV1. Indeed, 20 nM IGF-I potentiated pH currents to a similar degree as 1 μM insulin (Fig. 1g). Potentiation was dramatically reduced by the addition of tyrphostin A47 (100 μM; 10 min), a selective inhibitor for the insulin family of receptor tyrosine kinases (Fig. 1h). Together these data suggest that in oocytes, insulin and IGF-I are acting through IGFR to potentiate TRPV1 (Fig. 1i).

### Insulin and IGF-I potentiate native TRPV1 current in DRG neurons

**Figure 2:**
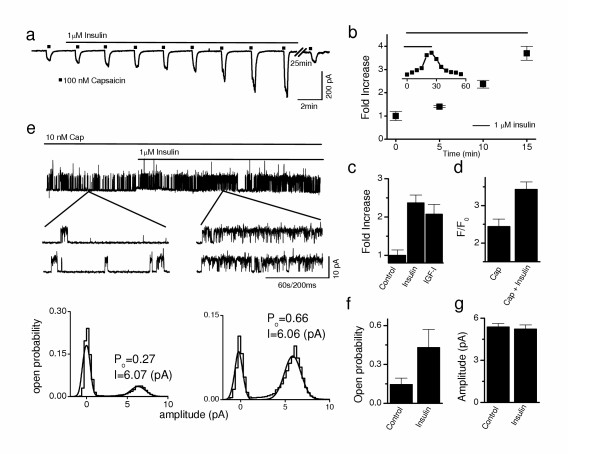
Insulin and IGF-I potentiate native TRPV1 currents in DRG neurons. **a**, Capsaicin (100 nM, applied at 2 min intervals) response was enhanced by insulin (1 μM) under perforated patch conditions. **b**, Time course of insulin induced potentiation of capsaicin currents (■: 500 nM capsaicin, 20 sec application) (*n *= 4, *P *< .01 at 5, 10, and 15 min incubation times). Inset shows the time course for experiment in Fig. 2a. **c**, Summary graph showing fold increase in TRPV1 currents following 10 min treatment with control (*n *= 15), insulin (1 μM; *n *= 11, *P *< .01) and IGF-I (20 nM; *n *= 4, *P *< .01). **d**, Under confocal microscopy, insulin (1 μM, 2 min) potentiated intracellular Ca^2+ ^rise in response to 500 nM capsaicin (*n *= 10, *P *< .05). **e**, Capsaicin induced single channel current activity recorded at +60 mV was increased by exposure of cell to insulin (1 μM). **f**, Mean capsaicin induced open probability (P_o_) in the absence and presence of insulin (*n *= 5, *P *< .05). **g**, Mean current amplitude observed from single channel recordings (*n *= 5).

Since insulin and IGF-I functions vary with cell type, it was important to establish that current potentiation also occurred in sensory neurons that express native TRPV1 [15,21]. Therefore, we determined the effect of insulin and IGF-I on capsaicin-induced TRPV1 responses using cultured DRG neurons. For whole cell recordings, the perforated patch technique was used to prevent desensitization and tachyphylaxis, minimize intracellular disruption, and maintain intact signaling cascades. In this setting, insulin (1 μM) induced a potentiation of the capsaicin (100 nM) response (Fig. 2a), which returned to control levels 20 minutes after its removal (Fig. 2b, inset). Like oocytes, similar results were seen with IGF-I, signifying that insulin was binding IGFR in DRG neurons (Fig. 2c).

To confirm electrophysiological experiments, measurements of Ca^2+^-uptake were performed using confocal microscopy (Fig. 2d). In DRG neurons, capsaicin (500 nM) produced a Ca^2+ ^influx that was increased in the presence of insulin. This shows that insulin elevated TRPV1-regulated Ca^2+ ^mobilization. Together this data, confirm that the potentiation seen with cloned TRPV1 in oocytes is present in native TRPV1 expressing peripheral sensory neurons as well.

### Insulin increases single channel activity

To understand the underlying molecular mechanism responsible for whole-cell potentiation, single channel currents were recorded in cell-attached patches from DRG neurons. Under this configuration, extracellular insulin would require receptor mediated signal transduction to facilitate changes in TRPV1 channel function recorded within the patch area (Fig. 2e). Channel open probability (P_o_), a measure of the time the channel spends in the open state, induced by capsaicin (10 nM) increased following bath-application of insulin (1 μM), outside the patch area (Fig. 2e, lower graphs). P_o _significantly changed from 0.15 ± 0.05 in control conditions to 0.43 ± 0.14 after insulin (Fig. 2f), without altering the single channel amplitude (Fig. 2g). Since insulin application was outside the patch, these data support intracellular signaling, as opposed to direct binding to TRPV1 as a mechanism for insulin-mediated potentiation.

### Signaling cascades utilized by Insulin and IGF-I

**Figure 3:**
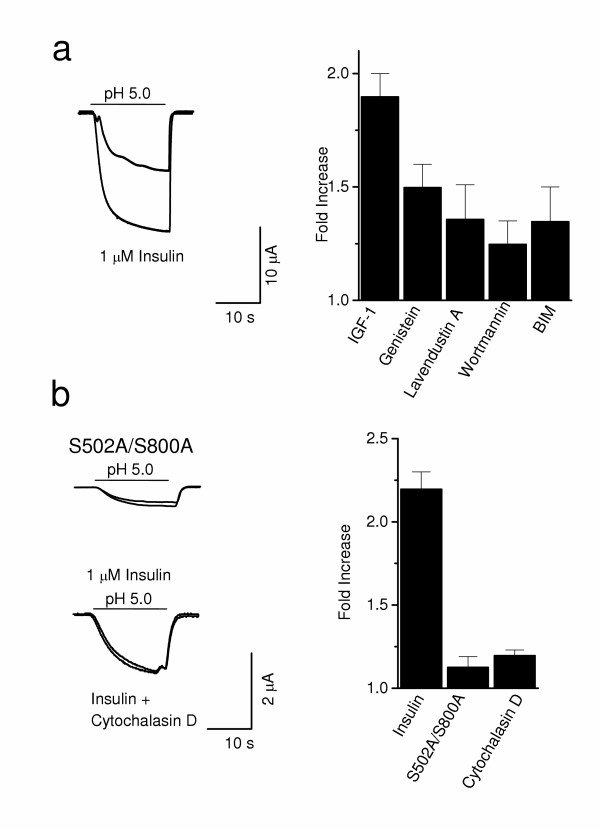
Singnaling cascades utilized by Insulin and IGF-I. **a**, Summary graph showing fold increase of current in the presence of IGF-I and the inhibitors: genistein (50 μM, *n *= 6, *P *< .05), lavendustin A (100 μM, *n *= 7, *P *< .05), wortmannin (100 nM, *n *= 5, P < .05) and BIM (200 nM, *n *= 5, *P *< .05). **b**, A PKC phosphorylation site TRPV1 mutant (S502A/S800A) (*n *= 6, *P *< .01, upper trace) and cytochalasin D (1 μM, *n *= 4, *P *< .01, lower trace) completely blocked insulin potentiation.

IR and IGFR produce their effects via an overlapping set of downstream enzymes [29], so we sought to identify signaling pathways involved in TRPV1 modulation. First, receptor tyrosine kinase (RTK) involvement was assessed by pretreating oocytes with membrane permeable inhibitors before and during insulin application (Fig. 3a). The nonspecific RTK blockers genistein (50 μM, 60 min) and lavendustin A (100 μM, 60 min) significantly reduced IGF-I (20 nM, 10 min) potentiation. Second, wortmannin (100 nM, 15 min), a specific phosphatidylinositol 3-kinase (PI(3)K) inhibitor, reduced potentiation as well. Thus, we reasoned that insulin and IGF-I utilize receptor tyrosine kinases to activate PI(3)K and prompt a signaling cascade that leads to TRPV1 current potentiation.

PI(3)K mediates some of its effects through various isoforms of protein kinase C (PKC) [29]. Therefore, we tested in oocytes expressing TRPV1 whether insulin and IGF-I could activate PKC, an enzyme known to potentiate TRPV1 through channel phosphorylation [8,31]. Bisindoylmaleimide (BIM; 200 nM, 60 min), a nonspecific PKC inhibitor, significantly decreased IGF-I potentiation (Fig. 3a). The importance of PKC was further demonstrated using a mutant TRPV1 (S502A/S800A), which lacks residues acting as substrates for PKC phosphorylation (31) (Fig. 3b, upper trace). Two consequences of these mutations were apparent. First, insulin/IGF-I potentiation was abolished. Second, current amplitude was smaller in oocytes injected with mutant TRPV1 compared to wild type, suggesting that phosphorylation might have an intrinsic effect on basal channel function. Thus, it appears that RTK, PI(3)K and PKC activation are required for current potentiation by insulin.

### Insulin and IGF-I translocate TRPV1 to the plasma membrane

**Figure 4:**
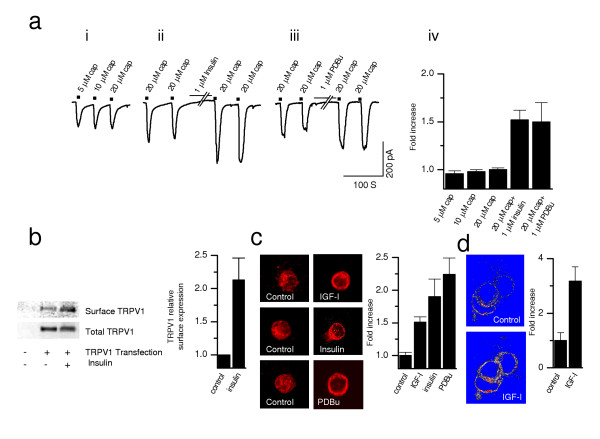
Insulin and IGF-I translocate TRPV1 to the plasma membrane. **a**, Potentiation of saturating concentrations of capsaicin response by insulin and PDBu. Application of 5, 10 and 20 μM capsaicin shows saturation of current response (**a_i_**), exposure (2–5 min) of insulin (**a_ii_**) or PDBu (**a_iii_**) caused a 50% increase in current amplitude induced by 20 μM capsaicin (**a_iv_**) (insulin n = 4 *P *< .01; PDBu n = 5 *P *> .01). **b**, Representative Western blot of surface protein and total TRPV1 from control and insulin-treated (10 μM, 15 min) HEK293 cells expressing TRPV1 (probed with anti-TRPV1 antibody). Surface represents fraction of biotinylated TRPV1 and total represents total amount of TRPV1 in immunoprecipitate. Quantitative analysis of insulin's effect on surface expression, with mean densities of surface bands normalized to control values for samples run on the same gel (*n *= 4, *P *< .01). **c**, Immunohistochemistry performed TRPV1 transfected HEK cells that were exposed to IGF-I (20 nM, 15 min), insulin (10 μM, 15 min) and PKC agonist, PDBu (10 μM, 15 min). Quantification of relative optical intensities (ROI, normalized as surface/cytosol for each cell): (control: *n *= 5; IGF-I: n = 7, *P *< .05; insulin: n = 3 *P *< .01; and PDBu: *n *= 7, *P *< .01). **d**, Confocal image showing a significant increase (3.17 ± 0.52 fold, *n *= 9; *P *< .01) in fluorescence intensity at the membrane 5 min after exposure to IGF-1 (50 nM) in HEK cells heterologously expressing GFP-TRPV1 fusion protein as compared to before IGF-1 application.

In a number of systems, insulin and/or IGF-I can increase surface content of effector molecules [32-34]. We have used five different approaches to examine whether TRPV1 translocation occurs in response to insulin or IGF-I. First, we used cytochalasin D (1 μM, 60 min) to inhibit actin polymerization and decrease vesicular fusion to the plasma membrane in oocytes (Fig. 3b, lower traces). Cytochalasin D almost completely blocked TRPV1 current potentiation by insulin, implicating involvement of protein trafficking affecting translocation of both TRPV1 and PKC. Second, insulin and PDBu not only increased the potency but also the efficacy of capsaicin induced currents. In DRG neurons, at saturating concentrations of capsaicin (20 μM) (Fig. 4a_*i*_) the current amplitude increased (>50%) following exposure (2–5 min) to insulin (1 μM) (Fig. 4a_*ii*_) or PDBu (1 μM) (Fig. 4a_*iii*_) suggesting recruitment of new channels into the plasma membrane or activation of previously silent channels (see also Fig. 1c). Third, Western blot analysis of cell-surface biotinylated TRPV1 expressed in HEK cells was carried out to test whether TRPV1 itself was being translocated to the plasma membrane (Fig. 4b). Analysis of band densities indicates that insulin (10 μM, 15 min) doubled surface TRPV1 expression levels relative to controls. Fourth, relative surface to cytosol optical intensities were quantified by immunofluorescence microscopy (Fig. 4c). These results, obtained with antibodies specific for TRPV1, show that IGF-I (20 nM, 15 min), insulin (10 μM, 15 min) and PDBu (10 μM, 15 min), all significantly increased surface TRPV1 expressed in HEK cells (Fig. 4c). Fifth, similar results were seen in HEK cells transiently transfected with green fluorescence protein (GFP)-tagged TRPV1. Exposure of IGF-I (50 nM) significantly increased the fluorescence intensity of the membrane within five minutes, indicating the accumulation of TRPV1 on the membrane (Fig. 4d). Together, these data suggest the involvement of receptor translocation in TRPV1 current potentiation and show that, in the presence of insulin, TRPV1 is mobilized to the plasma membrane from the cytosol.

## Discussion

From these results, we propose that insulin and its related growth factors provide both trophic and sensory support to peripheral nerve endings. Insulin and IGF-I can directly influence nociceptive ion channel function through phosphorylation and receptor translocation. In the presence of these modulators, TRPV1 is more responsive to painful stimuli (capsaicin, pH and heat) by increasing sensitivity and lowering thresholds. Based on this evidence, we conclude that insulin/IGF levels maintain TRPV1 function and their deficiency or resistance leads to deficits in inflammatory thermal sensation. The role of TRPV1 in animal models of diabetes has been suggested [27,35-37]. The intriguing aspect is that insulin deficiency results in thermal hyperalgesia in streptozotocin induced diabetes. Sensitization of TRPV1 by phosphorylation could account for the hyperalgesic phenotype seen in this animal model of diabetes [35]. The exact mechanism of sensitization of TRPV1 is not clear, but could be due to over compensation by other tropic factors, such as NGF in response to insulin deficiency or elevated PKC activity in diabetes [38].

Using multiple methods, we elucidate molecular mechanism(s) by which insulin/IGF-I potentiate TRPV1 current (Fig. 3 and 4). These neurotrophic factors, operating through RTKs, trigger a signaling cascade leading to PI(3)K and PKC activation [27,28,35]. PKC, an enzyme known to sensitize TRPV1 through phosphorylation [5,8,31], increases channel activity and receptor translocation to the cell surface [39]. Activation of a similar pathway to insulin by NGF via PI(3)K has been shown to robustly potentiate TRPV1 current [40]. Since insulin/IGF-I levels and PKC activity are altered in diabetes, we speculate that abnormalities in TRPV1 function may contribute to neuropathy in diabetes.

In DRG sensory neurons, diminished growth factor levels are some of the earliest changes noticed with diabetic neuropathy [11,20,21]. One explanation for diabetic neuropathy states that it is, in essence, a microvascular disturbance [41]. Peripheral C fibers (which express TRPV1) release vasoactive substances like CGRP, causing small vessel dilation to increase cutaneous circulation and nerve terminal viability. Activation of TRPV1 has been shown to induce and enhance the release of CGRP (7). This neural component of microcirculatory control is decreased in diabetic neuropathy, and the consequent reduction in local blood flow contributes to peripheral vascular complications [41,42]. With regards to growth factor deficiency, insulin has a vasodilatory effect that is dependent on CGRP release, which is compromised in diabetes [43,44]. Our findings illustrates insulin/IGFs can cause vasodilation via their influence on TRPV1.

A concept is emerging where signals emanating from IR and/or IGFR can activate kinases with the potential to control ion channel phosphorylation, subcellular localization and overall expression. Our work elucidates the mechanism insulin/IGF-I use for TRPV1 sensitization (i.e. RTK→PI(3)K→PKC), but the transduction pathway regulating expression has not been identified. Previously, it was suggested that insulin exerts influences on the same neurons that are responsive to NGF by sharing common pathways [13]. Recent reports demonstrate that NGF and glial cell line-derived neurotrophic factor (GDNF) upregulate TRPV1 expression on DRG neurons using transduction mechanisms common to insulin/IGF-I (i.e. MAPKs, PI(3)K, Ras, etc.) [11,40,45,46]. Taken together, these studies set precedence for growth factor influence on nociceptor levels and implicate signaling cascades, which may be compromised by the absence of insulin/IGF.

Insulin potentiates both whole cell and single channel currents mediated by NMDA receptors in *Xenopus *oocytes in a PKC-dependent manner [47]. Moreover, this effect was found to be due to membrane translocation involving both PI(3)K and PKC [33,34,38]. In DRG neurons insulin increased capsaicin induced cobalt uptake [48]. Along these same lines, insulin can potentiate currents in HEK293 cells through recruitment of GABA_A _receptors to postsynaptic domains [32]. In cultured cerebellar granular cells, IGF-I potentiates kinate receptors through a PI(3)K dependent mechanism [49].

Furthermore, vanilloid receptors (VRs) are present throughout the body, widely believed to have functions other than temperature sensation. TRPV1 expressed in the central terminals of the sensory neurons robustly modify synaptic transmission [50,51]. TRPV1 could be detected using RT-PCR technique throughout the neuroaxis [52] and identification of specific ligands such as NADA in certain brain regions confirms a role in the CNS [53]. The nature of the receptors involved in this response and their role in the CNS are not clearly understood, but suggestive of a direct or indirect role in modifying neurotransmitter release [54]. TRPV1 is also located in vasculature, bronchi and urinary bladder [7,55,56]. Modulation of these receptors by lack of insulin and IGF-I may contribute to CNS disturbances, cardiovascular, respiratory and urinary complications resulting from diabetes.

Lastly, these findings emphasize the importance of maintaining proper insulin levels and suggest a potential benefit of IGF-I administration in the treatment and prevention of diabetic complications. We propose that insulin and IGF-I therapy, partially working through TRPV1, can improve complications associated with diabetes mellitus.
